# A novel physiological feature selection method for emotional stress assessment based on emotional state transition

**DOI:** 10.3389/fnins.2023.1138091

**Published:** 2023-03-22

**Authors:** Zhen Li, Yun Xing, Yao Pi, Mingzhe Jiang, Lejun Zhang

**Affiliations:** ^1^The School of Electronic and Information Engineering, Tongji University, Shanghai, China; ^2^Institute of Automation, Chinese Academy of Sciences, Beijing, China; ^3^School of Biomedical Engineering, Sun Yat-sen University, Guangzhou, China; ^4^Cyberspace Institute Advanced Technology, Guangzhou University, Guangzhou, China; ^5^Research and Development Center for E-Learning, Ministry of Education, Beijing, China; ^6^College of Information Engineering, Yangzhou University, Yangzhou, China

**Keywords:** emotional stress, feature selection, electrocardiogram, galvanic skin resistance, blood volume pulse, emotional state transition

## Abstract

The connection between emotional states and physical health has attracted widespread attention. The emotional stress assessment can help healthcare professionals figure out the patient's engagement toward the diagnostic plan and optimize the rehabilitation program as feedback. It is of great significance to study the changes of physiological features in the process of emotional change and find out subset of one or several physiological features that can best represent the changes of psychological state in a statistical sense. Previous studies had used the differences in physiological features between discrete emotional states to select feature subsets. However, the emotional state of the human body is continuously changing. The conventional feature selection methods ignored the dynamic process of an individual's emotional stress in real life. Therefore, a dedicated experimental was conducted while three peripheral physiological signals, i.e., ElectroCardioGram (ECG), Galvanic Skin Resistance (GSR), and Blood Volume Pulse (BVP), were continuously acquired. This paper reported a novel feature selection method based on emotional state transition, the experimental results show that the number of physiological features selected by the proposed method in this paper is 13, including 5 features of ECG, 4 features of PPG and 4 features of GSR, respectively, which are superior to PCA method and conventional feature selection method based on discrete emotional states in terms of dimension reduction. The classification results show that the accuracy of the proposed method in emotion recognition based on ECG and PPG is higher than the other two methods. These results suggest that the proposed method can serve as a viable alternative to conventional feature selection methods, and emotional state transition deserves more attention to promote the development of stress assessment.

## 1. Introduction

Studies in the past decades have shown that emotions affect our physical and mental health (Cannon, 1939; Selye, 1975). It's of great significance to help healthcare professionals obtain the real experience of end-users on remote healthcare services, for the healthcare plan can be optimized according to the real feedback of patients. However, emotion recognition models based on facial expression are vulnerable to intentional deception. Instead, physiological signal features can truly reflect the characteristics of human emotional states, because they are not controlled by subjective consciousness, and are mainly related to the autonomic nervous system (ANS) and endocrine system (Ekman et al., 1983; Kreibig, 2010; Levenson, 2014). The transition of emotional state is reflected in people's physiological activities, such as stress will cause the increase of heart rate, respiratory rate (Ma et al., 2009), bronchiectasis, pupil dilation, skin sweating, and other symptoms (Pagani et al., 1991; Arza et al., 2019; Greco et al., 2021). Considering the above factors and the needs (weak interference and easy acquisition) of wearable devices, ECG signal, GSR signal, and BVP signal are selected as the collected physiological signals in this study.

For the research status of psychological stress recognition in recent years, Balamurali et al. proposed a model using respiratory rate, ECG, and GSR for automatic and intelligent anxiety detection, they compared the basic machine learning algorithm and the ensemble-based classification algorithm, and the results showed that the binary classification accuracies of the extension number, random forest and bagged decision tree were the highest (80%) (Balamurali et al., 2022). Pourmohammadi and Maleki recruited 34 healthy participants for continuous personalized stress detection utilizing a combination of ECG and EMG signals as input. The method they proposed is a fuzzy model that is highly correlated with perceived pressure. The model includes fuzzy inference system and fuzzy clustering algorithms. The result showed that there was a strong correlation between pressure and perceived pressure, with a specific correlation coefficient of 0.959 (Pourmohammadi and Maleki, 2021). Azam et al. built an ensemble classifier with 12 selected PPG features to detect social stress, and achieved an accuracy of 92.8%, and also showed that the median frequency and average frequency are the most effective features for stress detection (Azam et al., 2021). Zheng et al. used stimuli (video clips) to induce psychological stress, collected EMG signals from 25 healthy subjects, and extracted Simple Square Integral (SSI), Integral EMG (IEMG), waveform length from EMG signals (WL), and absolute standard deviation (DASDV) 4 time-domain features, using KNN and FKNN two non-linear classification algorithms to classify psychological stress and non-psychological stress, the maximum recognition accuracy is 70.85%, where WL is the feature with the biggest significant difference (Zheng et al., 2013). Shon et al. conducted research on the detection of psychological stress state-based on EEG signals, used the genetic algorithm to effectively select the features, and compared the proposed method with the feature selection method of principal component analysis. The research results showed that the accuracy of the proposed method is about 6% higher than the accuracy of principal component analysis (Shon et al., 2018). In order to achieve the purpose of continuous monitoring of different emotions, Naji et al. extracted features with sliding window technology (customized window and step size) (Han et al., 2017). Nigam et al. (2021) and Zhang et al. (2021) achieved good emotion recognition performance using deep learning algorithms. Han et al. simulated the stress conditions in an office work environment. This simulated stress environment consists of mental stress and psychosocial stress. Their study used ECG and RSP signals as input, and their proposed model combined the random forest and the support vector machine. The accuracy of their model in the three-classification task is 84%, and the accuracy in the binary classification task is 94% (Han et al., 2017).

At present, more and more researchers have begun to use deep learning models to recognition emotion and have achieved better results on public data sets. However, advantages of deep learning algorithms rely on big data, and the deep learning algorithms are lack of interpretability. Finding the sensitive physiological feature subset of the corresponding state can not only improve the accuracy of the recognition model, but also ensure the stability and interpretability of the model (Tang et al., 2018, 2019). Therefore, this paper conducted research on psychological stress recognition based on conventional feature engineering methods. On the other hand, the existing studies use the difference of physiological features between discrete emotional states to select feature subsets. However, the emotional state of the human body is dynamic and continuously changing,and the time interval between different emotional states may lead to changes in physiological signals and ultimately affect the selection of sensitive features. The conventional feature select method ignored the change process of individual psychological stress in real life. Psychological research shows that under the continuous action of strong psychological stress, the receptivity of the receptor decreases. Therefore, this study put forward the hypothesis that the changes of physiological features associated with psychological stress are more significant before and after the emotion transition. Based on the above assumptions, this study focused on the changes of physiological features of emotional state transition, which is closer to the real response in the real life, so as to achieve more accurate psychological stress detection.

Aiming at the above problems existing in the current emotional-aware systems, this paper proposed a novel feature selection method based on emotional state transition. The specific research contents of this paper include:

The contributions of this work:

- A dedicated experimental protocol was designed and implemented where specific affective valence levels are elicited by images selected from the IAPS, while three peripheral physiological signals, i.e., ElectroCardioGram (ECG), Galvanic Skin Resistance (GSR), and Blood Volume Pulse (BVP) are acquired from 85 subjects simultaneously.
- A feature selection method based on emotional state transition were proposed and compared with conventional methods.

## 2. Methods and materials

### 2.1. Experimental protocol and data collection

#### 2.1.1. Stimuli

**Figure 1:**
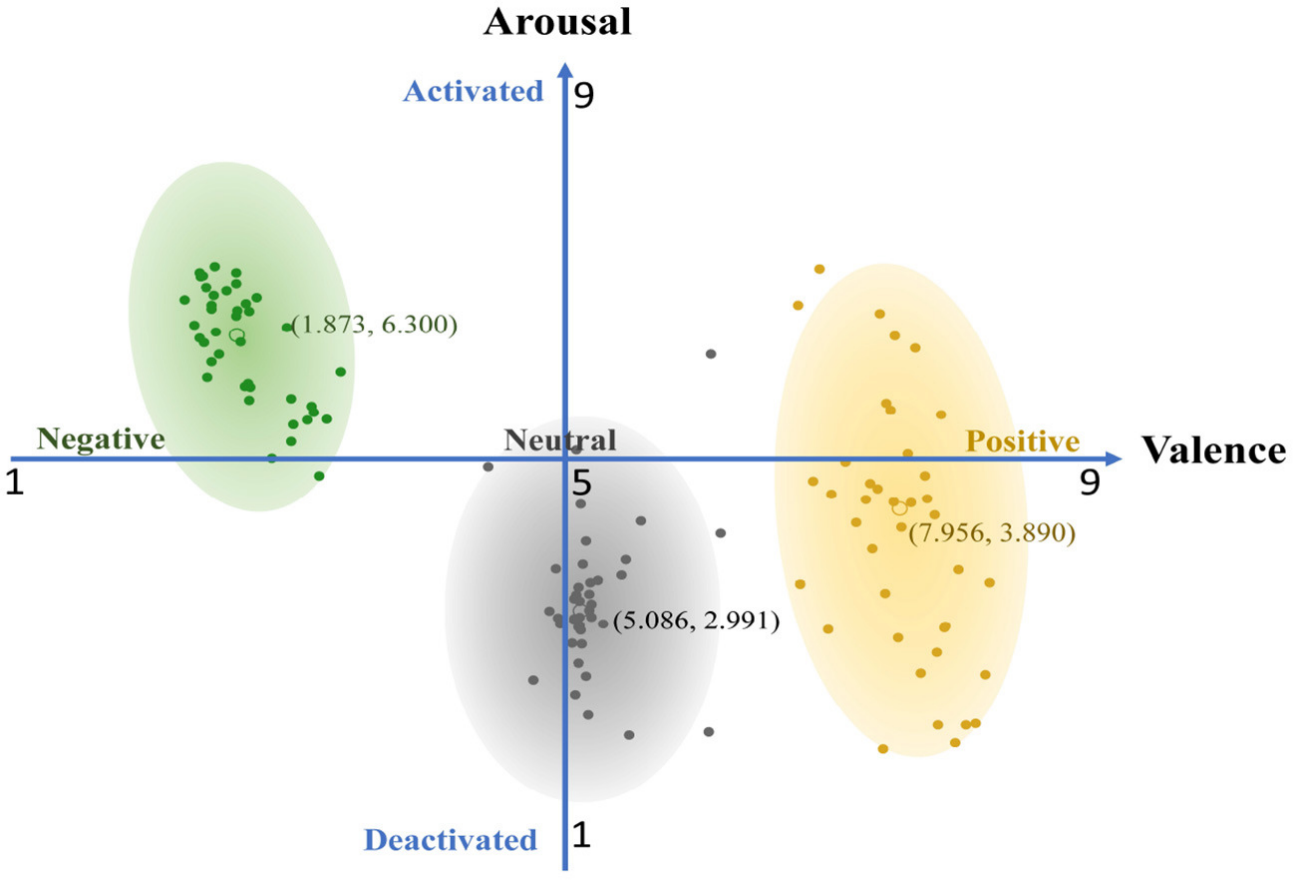
The valence levels and arousal levels of stimulus.

One hundred and twenty images were manually selected from the IAPS (Lang et al., 1997) and divided into three groups according to valence levels. The IAPS is a set of standardized emotional stimulation picture systems developed by the National Institute of Mental Health (NIMH) to provide stimulating materials for emotion research. IAPS currently has more than 900 images, most of the photos are collected from newspapers, magazines, and other media, with clear content and resolution, including various human emotions or various objects. These photos have advantages of good test-retest reliability and internal consistency, and pictures of IAPS have been shown to induce positive, negative, or neutral affective states. The pictures of IAPS are attached with the values of arousal, valence, and dominance assessed by the researchers according to the SAM scale (9-point scale). In the emotion model, the ratings of valence dimension are used to differentiate positive (pleasant) from negative (unpleasant) emotional states, and the ratings of arousal dimension are used to differentiate activated (excited) from deactivated (relaxed) emotional states. In this study, inducing images are selected based on valence ratings provided by IAPS. 40 pictures with low valence level (mean: 1.873, std: 0.403) are selected as stimuli of negative emotion, and 40 pictures with middle valence level (mean: 5.086, std: 0.393) are selected as stimuli of neutral emotion, 40 pictures with high valence level (mean: 7.956, std: 0.466) are selected as stimuli of positive emotion. The specific valence levels and arousal levels distribution of 120 stimulating pictures are shown in Figure 1.

#### 2.1.2. Participants

Eighty-five healthy university students (39 males and 46 females) with an average age of 23.1 ±2.4 were recruited in this study. All volunteers had no history of heart disorders, musculoskeletal disorders, chronic diseases, or mental diseases, and their Body Mass Index (BMI) was lower than 28. All participants were told not to exercise vigorously, smoke, drink coffee or alcohol within 24 h before the start of the experiment, and they were asked to sign informed consent before experiment.

#### 2.1.3. Experimental setup

**Figure 2:**
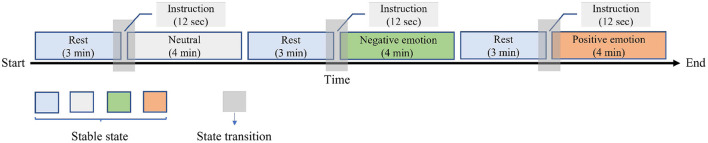
Data collection protocol.

**Figure 3:**
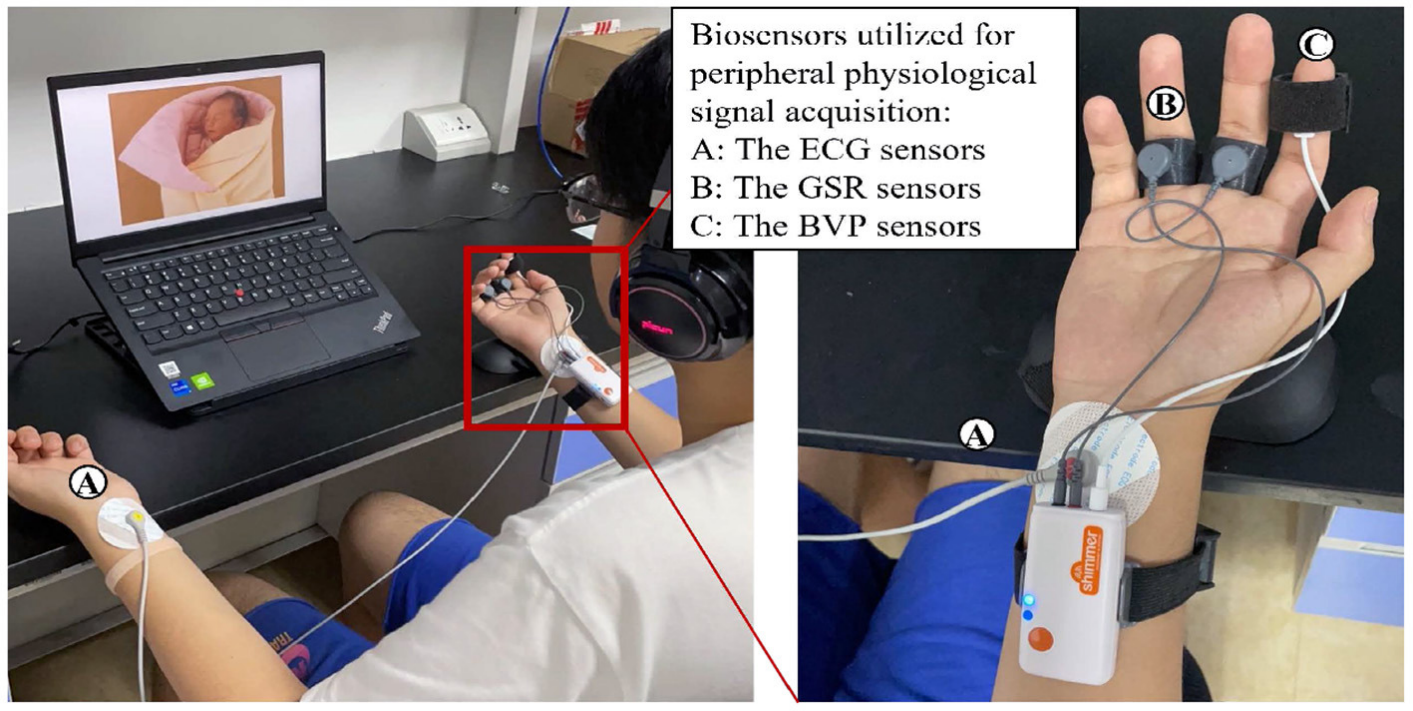
Participant wears three bio-sensors to collect physiological signals.

Figure 2 illustrates the protocol and experiment procedure. The experiment was conducted in a quiet environment with controlled lighting and temperature (24±2°*C*). Before the beginning of the experiment, all subjects were informed of the next experimental process and that they have rights to withdraw from the experiment unconditionally at any time., and all subjects were asked to lean back on the lounge chair and keep a comfortable position during the whole experiment. Then, the researchers placed corresponding sensors at the wrist, right index finger, right middle finger, and right ring finger of the subjects to collect electrocardiogram (ECG), blood volume pulse (BVP), and galvanic skin resistance (GSR) signals, respectively (see Figure 3). The whole experimental process mainly includes three emotion-inducing parts. In the first part, play 3-min light music (Pachelbel's Canon in D) to make the subject relax, and then 40 neutral pictures selected from the IAPS were presented on the computer screen to stimuli neutral emotional responses. Each picture was presented for 6 s, and the total time of evoking the emotional process was 4 min. In the second part, play 3-min of light music to make the subjects relax, and then play 40 negative pictures selected from the IAPS on the computer screen to stimuli negative emotional responses. Each picture is presented for 6 s. In the third part, play 3-min of light music to make the subject relax again, then play 40 positive pictures selected from IAPS to stimuli positive emotional responses. ECG, BVP, and RSR signals were collected throughout the experiment for each participant. The sampling rate was 400 Hz for ECG, and the sampling rates of BVP and GSR were both 201 Hz.

### 2.2. Analysis of psychophysiological signals

**Figure 4:**
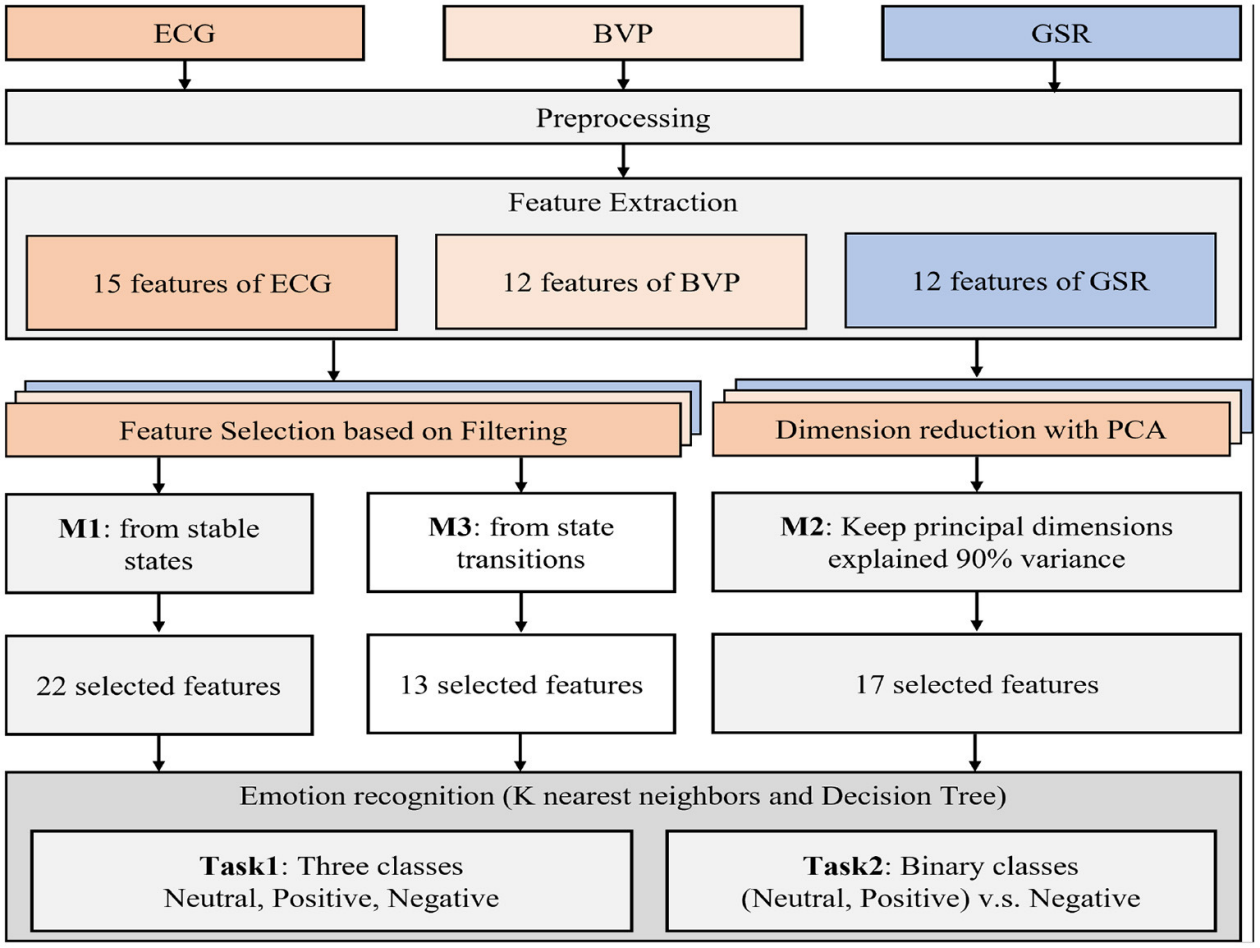
Processing flow of physiological signals.

The signals processing flow of this paper is shown in Figure 4. A preprocessing step is employed to remove interference and noise of three physiological signals. After preprocessing, 39 features were extracted. Then, the conventional feature selection method, PCA method, and the feature selection method based on emotion state transition were used to select significant features from the extracted physiological features, respectively. The selected features were input to train the K-Nearest Neighbors (KNN) classifier, the Decision Tree (DT) classifier, the Random Forest (RF) classifier, and the Support Vector Machine (SVM) classifier, and the performance of these three feature selection methods is compared and analyzed.

#### 2.2.1. Preprocessing

The Shimmer3 GSR+ Unit was used to collect PPG and GSR signals, while the device of Tempest Couriers was used to collect ECG signals. The data file collected by the Shimmer sensor provides the timestamp information of each data point. Therefore, in the experiment, the start time was recorded and data synchronization between different devices was achieved based on the timestamp and the start time. In the stage of signal preprocessing. Firstly, a Butterworth band-pass filter with cut-off frequencies of 1–40 Hz was used to remove noise and baseline wander of raw ECG signal and raw BVP signal. Next a notch filter was utilized to reduce 50 Hz power frequency interference. Secondly, the GSR signal is downsampled to 4 Hz, and then the signal is decomposed by cvxEDA., and the size of the sliding window is 60 s and the step size is 1 s. Thirdly, we calculated the duration between the two R waves (R–R interval) in ECG and the peak of the BVP to obtain useful information.

#### 2.2.2. Feature extraction

In this section, a total of 39 physiological features (including 15 ECG features, 12 BVP features, and 12 GSR features) were extracted to make a feature selection performance comparison among the proposed method and two conventional methods.

For time-domain analysis of ECG, the mean value of RR intervals (*MEANRR*_*ECG*_) was employed as the first index, and the second index was the standard deviation of the RR intervals (*SDNN*_*ECG*_) (Shi et al., 2017). The *RMSSD*_*ECG*_ represents the square root of the mean squared differences of successive RR intervals. The *PNN*50_*ECG*_ was defined as the proportion of differences between successive RR intervals greater than 50 ms whereas *NN*50_*ECG*_ was defined as the mean of RR intervals number of pairs of successive NN (R-R) intervals that differ by more than 50 ms., and the HR was defined as the number of contractions of the heart per minute. For frequency-domain analysis of ECG, the heart rate variability (HRV) spectrum was decomposed into three separate frequency bands: a very low-frequency band (*VLF*_*ECG*_) with spectral components below 0.04 Hz, a low-frequency band (*LF*_*ECG*_) with spectral components from 0.04 to 0.15 Hz and a high-frequency band (*HF*_*ECG*_) comprising frequencies from 0.15 to 0.4 Hz, and *LF*/*HF*_*ECG*_ means the ratio of *LF*_*ECG*_ power to *HF*_*ECG*_ power. For non-linear indices of ECG, multiscale entropy (*MSE*_*ECG*_) presents the feature of the complexity of fluctuations over a range of time scales and is employed as an extension of standard sample entropy measures, hence the multiscale entropy with the scale of 1~ 5 were calculated.

Photoplethysmography (PPG) is an optical non-invasive method measuring variations of skin hue associated with concurrent changes in blood volume in subcutaneous blood vessels during the cardiac cycle (Korhonen and Yli-Hankala, 2009). From the BVP signals the pulse rate (*PR*_*BVP*_), the standard deviation of normal-to-normal peak (*SDNN*_*BVP*_), root mean square of normal-to-normal peak (*RMSSD*_*BVP*_), mean of inter-beat-intervals (*MEANRR*_*BVP*_), power frequency band of below 0.15 Hz (*LF*_*BVP*_), the power frequency band of 0.15–0.40 Hz (*HF*_*BVP*_), the time interval between two consecutive pulse onsets (*WIDTH*_*BVP*_), the amplitude difference between the pulse peak and the pulse onset (*H*_*W*_*BVP*_) were extracted. Peripheral vasoconstriction can also monitor stress, and the pulse wave amplitude (*HIGH*_*BVP*_) decreases when stress occurs (Giannakakis et al., 2019). Therefore, the maximum value of pulse wave amplitude (*MAXPA*_*BVP*_), the standard deviation of pulse wave amplitude (*STDPA*_*BVP*_), and mean of pulse wave amplitude (*MEANPA*_*BVP*_) were also calculated.

GSR is a physiological measurement of electricity flow through the skin. Through the monitoring of skin conductivity, the situation of skin sweating can be indirectly acquired (Montagu and Coles, 1966). For skin conductance level (SCL) of GSR, the average value of the tonic component (*MEANT*_*GSR*_), the standard deviation of the tonic component (*STDT*_*GSR*_), and area under the curve of the tonic component (*AUCT*_*GSR*_) were extracted as features of GSR. For skin conductance response (SCR) of GSR, the standard deviation of the phasic component (*STDR*_*GSR*_), area under the curve of the phasic component (*AUCP*_*GSR*_), number of peaks of the phasic component (*NUMP*_*GSR*_), the maximum value of peak amplitude of the phasic component (*MAXPA*_*GSR*_), mean of the peak amplitude of the phasic component (*MEANPA*_*GSR*_), the standard deviation of the peak amplitude of the phasic component (*STDPA*_*GSR*_), the average value of the rise time (*MEANPR*_*GSR*_), the standard deviation of the rise time (*STDPR*_*GSR*_), and power spectral density of GSR (*PSD*_*GSR*_) were extracted.

### 2.3. Feature selection methods

The main task of feature selection is to select the important features from the original feature set, reduce the number of features, and preserve the classification information as much as possible. The feature selection method will directly influence the performance of classification models. The existing feature selection methods still can not select the optimal feature subset of emotion recognition. Therefore, a new feature selection method was proposed. In this section, two generally applied feature selection methods and the proposed method are introduced.

#### 2.3.1. Feature selection method based on discrete emotional states

For the method based on discrete emotional states, the first step is to select the features that offer contributing information for the classification task applying the Kruskal-Wallis test for paired samples. Next, if the Pearson correlation coefficient between a newly selected feature and any feature in the initial set is higher than 0.9, the new feature will be discarded (Montesinos et al., 2019). Then, the initially selected features will be input into the classifier, and the final feature selection will be carried out according to the classification performance of different feature combinations. To be noted, the existing methods mainly consider whether the features of discrete emotional states are significantly different, ignoring that emotion is a state process that changes with time, which may lead to the loss of important features.

#### 2.3.2. Feature selection method based on PCA

PCA was first proposed by Pearson in 1994 and turned out to be one of the classical methods of mathematical-statistical analysis, feature selection, and dimension reduction. Principal component analysis (PCA) projects multivariate data into a new low dimensional coordinate system through orthogonal linear transformation such that the maximum variance in the data corresponds to the first coordinate system and the minimum variance in the data corresponds to the last coordinate system (Abdi and Williams, 2010). PCA is used to eliminate the correlation between variables by assuming the correlation is linear, hence PCA might don't work for non-linear correlation. PCA assumes that the variables follow Gaussian distribution. When the variables do not follow a normal distribution (such as uniform distribution), scaling down and rotation will occur.

#### 2.3.3. Feature selection method based on emotional state transition

**Figure 5:**
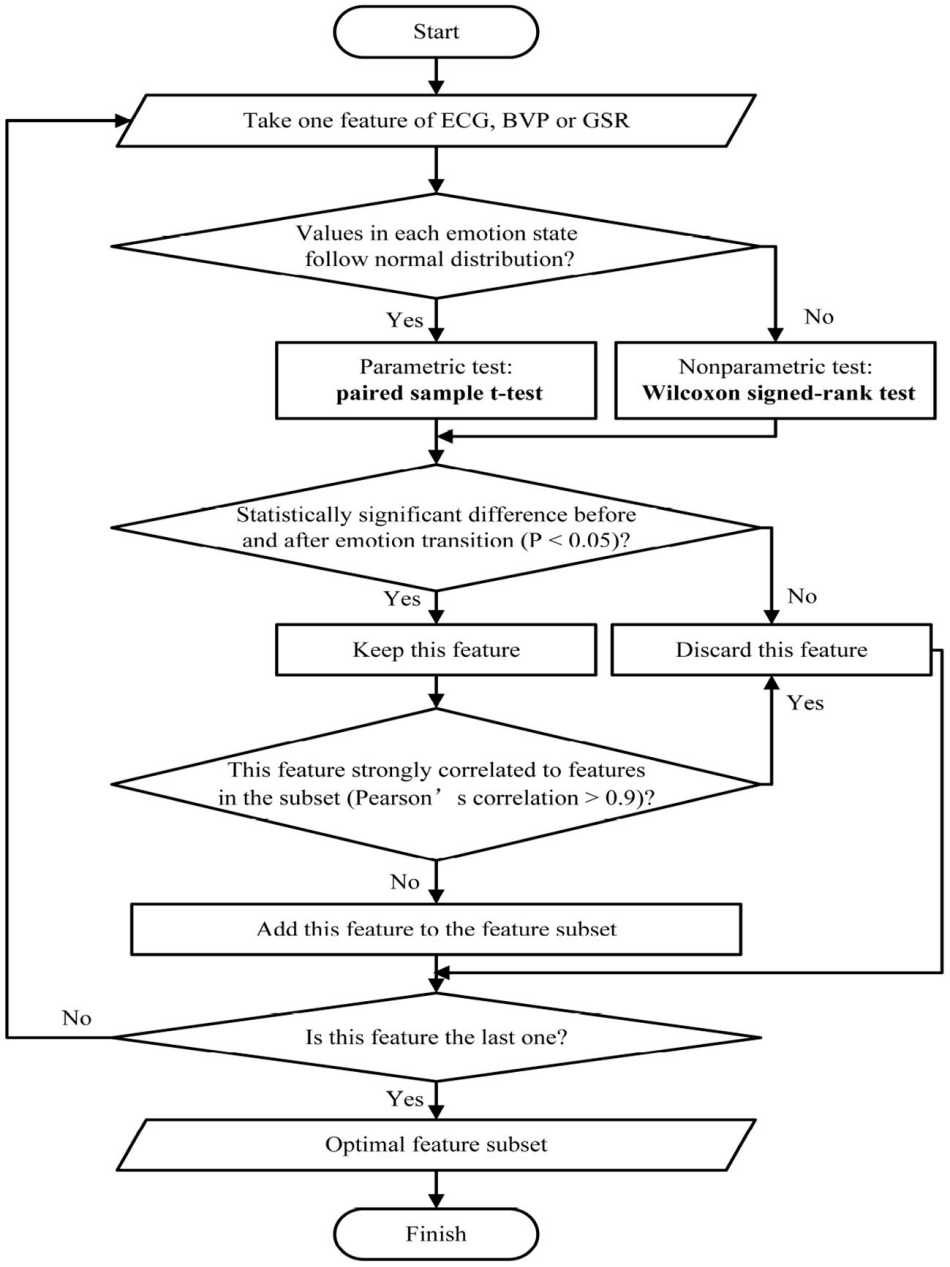
Flowchart of the proposed feature selection method.

Figure 5 shows the flowchart of the proposed feature selection method. For each extracted physiological signal feature. If the feature value follows the normal distribution, a parametric test (paired sample *t*-test) will be conducted. If not, a non-parametric test (Wilcoxon signedrank test) will be conducted. Afterward, the features with significant differences before and after each emotional state transition will be selected. If the Pearson correlation coefficient of the newly selected feature and any feature in the subset is greater than 0.9, this feature will be discarded, the whole process will end until all the extracted features are screened. Finally, the optimal feature subset is built.

## 3. Experiments and results

In this section, the emotion models based on machine learning algorithms are built and evaluated utilizing MathWorks's Statistics and Machine Learning Toolbox in Matlab R2018a., and SPSS software (Ver. 20, IBM, USA) was used to perform all statistical analyses in this paper.

### 3.1. Feature selection results

As mentioned above, two classical feature selection methods and the proposed method were applied to analyze all the extracted features for building the optimal feature subset. The feature selection results of method 1 and method 3 are listed in Table 1, where the sign “*” in Table 1 represents the *P*-value is less than 0.05.

**Table 1:** Selection results of features.

**Signal**	**Feature**	**Method 1 (M1), mean of feature value**				**Method 3 (M3), mean of feature value**			
		**Neutral**	**Negative**	**Positive**	***P*** ** < 0.05**	**Neutral** ~**negative**	**Negative**~**neutral**	**Neutral**~**positive**	***P*** ** < 0.05**
ECG	*SDNN*_*ECG*_	0.047	0.046	0.044	*	0.046/0.052	0.044/0.053	0.045/0.042	*
	*RMSSD*_*ECG*_	0.044	0.044	0.039	*	0.045/0.045	0.042/0.043	0.038/0.036	—
	*MEANRR*_*ECG*_	0.787	0.786	0.777	*(—)	0.790/0.782	0.783/0.776	0.764/0.776	—
	*NN*50_*ECG*_	11.665	12.956	10.539	*	11.433/14.265	11.747/11.578	10.798/10.699	*
	*PNN*50_*ECG*_	0.164	0.183	0.140	*(—)	0.163/0.194	0.164/0.160	0.136/0.140	—
	*MSE*1_*ECG*_	1.806	1.785	1.748	—	1.762/1.737	1.778/1.643	1.648/1.725	—
	*MSE*2_*ECG*_	1.890	1.866	1.885	—	1.864/1.803	1.937/1.762	1.758/1.852	—
	*MSE*3_*ECG*_	1.714	1.668	1.753	*	1.694/1.725	1.633/1.587	1.686/1.735	—
	*MSE*4_*ECG*_	0.674	0.634	0.738	*	0.706/0.512	0.691/0.719	0.705/0.758	—
	*MSE*5_*ECG*_	0.048	0.075	0.046	—	0.047/0.077	0.103/0.054	0.045/0.060	—
	*HR*_*ECG*_	80.756	81.060	81.922	*(—)	79.041/85.364	80.155/81.128	83.646/83.109	*
	*VLF*_*ECG*_	0.793	0.788	0.781	*	0.800/0.778	0.783/0.785	0.765/0.774	—
	*LF*_*ECG*_	2.295	2.285	2.278	*(—)	2.303/2.271	2.281/2.282	2.254/2.266	*
	*HF*_*ECG*_	2.271	2.239	2.232	*(—)	2.285/2.209	2.233/2.233	2.176/0.918	*(—)
	*LF*/*HF*_*ECG*_	1.028	0.997	1.067	*	1.029/1.058	1.008/0.986	0.918/0.977	*
BVP	*SDNN*_*BVP*_	0.052	0.058	0.054	*	0.048/0.0586	0.056/0.063	0.059/0.056	*
	*RMSSD*_*BVP*_	0.052	0.060	0.053	*	0.049/0.061	0.057/0.057	0.056/0.054	*
	*MEANRR*_*BVP*_	0.780	0.782	0.779	*(—)	0.778/0.776	0.783/0.776	0.768/0.776	—
	*LF*_*BVP*_	−0.097	−0.091	−0.089	*	−0.094/−0.094	−0.092/−0.090	−0.091/−0.090	—
	*HF*_*BVP*_	−2.735	−2.758	−2.728	—	−2.741/−2.754	−2.749/−2.741	−2.722/−2.740	—
	*WIDTH*_*BVP*_	0.007	0.008	0.007	—	0.007/0.009	0.007/0.007	0.008/0.008	—
	*HIGH*_*BVP*_	0.132	0.134	0.147	*	0.138/0.151	0.137/0.163	0.165/0.150	—
	*H*_*W*_*BVP*_	11.643	9.591	10.470	*	12.313/8.270	9.979/9.901	10.151/9.832	*
	*MAXPA*_*BVP*_	0.881	0.816	0.885	*	0.920/0.769	0.849/0.835	0.851/0.848	*
	*STDPA*_*BVP*_	0.094	0.100	0.107	*(—)	0.096/0.115	0.102/0.117	0.124/0.111	—
	*MEANPA*_*BVP*_	0.722	0.623	0.705	*(—)	0.761/0.544	0.648/0.624	0.647/0.653	*(—)
	*MEANRR*_*BVP*_	79.054	79.214	79.361	*(—)	79.163/79.786	79.035/79.322	80.210/79.408	—
GSR	*MEANT*_*GSR*_	−0.135	0.093	−0.406	*	−0.724/−0.021	0.082/0.319	−0.157/0.087	*
	*STDT*_*GSR*_	0.200	0.227	0.196	—	0.148/0.346	0.216/0.322	0.218/0.255	—
	*AUCT*_*GSR*_	−55.086	1.026	−31.418	*	−39.036/42.155	−0.383/−17.245	−20.940/−55.341	—
	*STDR*_*GSR*_	0.108	0.272	0.145	*	0.092/0.368	0.243/0.394	0.243/0.220	*
	*AUCR*_*GSR*_	−3.475	−6.607	3.511	*	22.190/−35.097	−9.232/−26.143	7.429/−13.874	—
	*NUMP*_*GSR*_	24.394	39.248	24.170	*	21.771/42.145	39.229/37.855	32.181/29.024	—
	*MAXPA*_*GSR*_	2.192	5.479	2.775	*	1.846/7.109	4.468/6.450	4.449/3.657	*
	*MEANPA*_*GSR*_	0.372	0.907	0.424	*(—)	0.299/1.184	0.768/0.967	0.608/0.526	*(—)
	*STDPA*_*GSR*_	0.472	1.163	0.586	*(—)	0.377/1.479	0.981/1.363	0.867/0.771	*(—)
	*MEANPR*_*GSR*_	1.002	0.685	0.885	*	0.905/0.651	0.607/0.679	0.711/0.788	—
	*STDPR*_*GSR*_	0.829	0.607	0.719	*	0.799/0.569	0.579/0.584	0.633/0.724	*
	*PSD*_*GSR*_	3.777	13.366	5.116	*	5.491/12.204	9.096/8.068	2.999/3.660	—

For method 1, the Kruskal-Wallis test was employed to analyze the significant difference between different emotional states (Neutral emotion vs. Negative emotion, Negative emotion vs. Positive emotion, and Positive emotion VS Neutral emotion), and one feature will be selected if the *p* < 0.05 in the above three cases. Twelve ECG features, ten BVP features, and eleven GSR features were initially selected by M1. After Pearson correlation analysis, 11 features were discarded. Then, 7 ECG features were selected from 15 extracted features, are *SDNN*_*ECG*_, *RMSSD*_*ECG*_, *NN*50_*ECG*_, *MSE*3_*ECG*_, *MSE*4_*ECG*_, *VLF*_*ECG*_, and *LF*/*HF*_*ECG*_, respectively. Six BVP features were selected from 12 extracted features, are *SDNN*_*BVP*_, *RMSSD*_*BVP*_, *HIGH*_*BVP*_, *LF*_*BVP*_, *H*_*W*_*BVP*_, and *MAXPA*_*BVP*_, respectively. Nine GSR features were selected from 12 extracted features, they are *MEANT*_*GSR*_, *AUCT*_*GSR*_, *STDPA*_*GSR*_, *AUCP*_*GSR*_, *NUMP*_*GSR*_, *MAXPA*_*GSR*_, *MEANPR*_*GSR*_, *STDPR*_*GSR*_, and *PSD*_*GSR*_, respectively. Hence, a total of 22 features were selected by applying method 1, and the dimension of selected features is 22 (categories of feature) × 181 (number of each feature) × 3 (emotion states) × 85 (subjects).

For method 2, PCA obtains the eigenvectors and eigenvalues by decentralizing the original features and solving the covariance matrix. In this study, the dimension of the original features for each subject and each emotional state is 39 × 181, whereas the feature dimension after dimension reduction is 17 × 181, and the corresponding dimension of the ECG feature is 6 × 181, the dimension of the BVP feature is 5 × 181, the dimension of the GSR feature is 6 × 181.

For method 3, the parametric test (paired sample *t*-test) or the non-parametric test (Wilcoxon signed-rank test) was applied to examine the significant difference before and after emotional state transition (Neutral emotion to Negative emotion, Negative emotion to Neutral emotion, and Neutral emotion to Positive emotion), and one feature will be selected if the *p* < 0.05 in the above three cases. Then, 6 ECG features were selected, are *SDNN*_*ECG*_, *NN*50_*ECG*_, *HR*_*ECG*_, *LF*_*ECG*_, *HF*_*ECG*_, and *LF*/*HF*_*ECG*_, respectively. Five BVP features were selected, they are *SDNN*_*BVP*_, *RMSSD*_*BVP*_, *H*_*W*_*BVP*_, *MAXPA*_*BVP*_, and *MEANPA*_*BVP*_, respectively. Six GSR features were selected, they are *MEANT*_*GSR*_, *STDR*_*GSR*_, *MAXPA*_*GSR*_, *MEANPA*_*GSR*_, *STDPA*_*GSR*_, and *STDPR*_*GSR*_, respectively. Whereas, *HF*_*ECG*_, *MEANPA*_*BVP*_, *STDPR*_*GSR*_, and *STDPA*_*GSR*_ were discarded because they are strongly correlated to features in the subset [“*(—)” in Table 1 means the *P* < 0.05 and the Pearson's correlation coefficient > 0.9]. Hence, 13 features were finally selected by applying method 3, and the dimension of selected features is 13 (categories of feature) × 181 (number of each feature) × 3 (emotion states) × 85 (subjects).

### 3.2. Performance of selected features in emotion recognition

**Figure 6:**
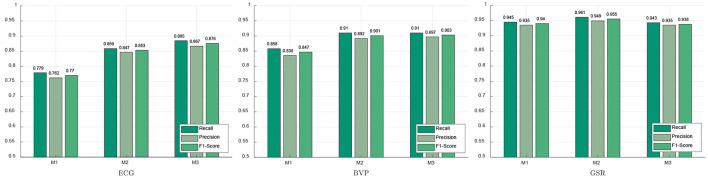
Performance of different feature selection methods in emotion recognition.

The experimental results of the performance of three feature selection methods in emotion recognition (negative emotion / neutral emotion / positive emotion) are shown in Figure 6. It can be concluded that the feature selection method based on PCA (M2) achieved the worst performance on the emotion recognition task. While the Recall, Precision, and F1-Score of the proposed feature selection method (M3) are highest in ECG and BVP, are 0.885, 0.867, 0.876 and 0.910, 0.897, 0.903, respectively, indicating that heart rate variability and pulse rate variability indicators are more obvious for emotion recognition.

However, the GSR signal in Figure 6 has a better classification performance based on method 1 (M1). The evaluation indicators of the proposed method (M3) are 0.943 (Recall), 0.935 (Precision), and 0.938 (F1-score), respectively. From these results above, it can be concluded that the proposed method (M3) is better in ECG and PPG, probably because most of the features extracted by ECG and PPG are consistent with HRV and PRV. To be noted, HRV and PRV change obviously when the individual's emotion fluctuates, especially when experiencing psychological stress.

### 3.3. Performance of selected features in psychological stress detection

**Figure 7:**
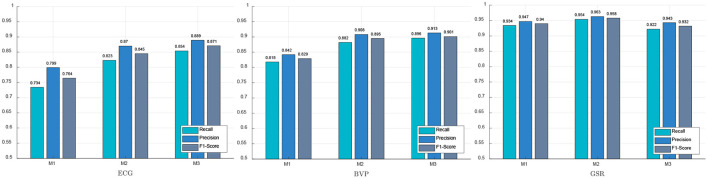
Performance of different feature selection methods in psychological stress detection.

In this section, the negative emotion is considered as psychological stress, while both the neutral emotion and the positive emotion are considered as the non-stress state. Four classifiers (KNN, DT, RF, and SVM) are adopted to classify psychological stress state and non-stress state, the experimental results of performance of three feature selection methods on psychological stress detection are shown in Figure 7. It can be seen that the feature selection method based on PCA (M2) achieved the worst performance on the psychological stress detection task. The Recall, Precision, and F1-Score of the proposed method (M3) in ECG and PPG signals are the highest, which are 0.854, 0.889, 0.871 and 0.896, 0.913, 0.901, respectively. However, method 1 (M1) achieved the best performance on the GSR signal, while the Recall, Precision, and F1-Score are 0.954, 0.963, and 0.958, respectively. It can also be concluded that the feature selection method based on emotional state transition achieved the best performance on psychological stress detection in ECG signals and pulse wave signals.

## 4. Discussion

This study aimed to optimize psychological stress detection systems based on ECG, BVP, and GSR signals. After collecting three different physiological signals from 85 healthy subjects while watching three groups of images with different valence levels, an efficient feature selection method for emotion recognition was proposed based on emotional state transition. Table 1 showed the results of feature selection using M1 and M3. The 17 features preliminarily selected in method 3 are subsets of the 33 features preliminarily selected in method 1. After Pearson correlation analysis, method 1 discarded 11 features, and method 3 discarded 4 features. It can be concluded that the M3 can select the most important features efficiently.

Four widely applied classification algorithms (KNN, DT, RF, and SVM) were used to analyze the performance of two conventional feature selection methods and the proposed method. In three emotions classification models based on 10-fold cross-validation, the proposed feature selection method can achieve better performance compared to conventional methods with fewer ECG features or BVP features.

It is of great significance to build a psychological stress detection system that can be applied to real life (Gjoreski et al., 2016; Castaldo et al., 2019). Hence, we built binary classification models (stress, non-stress). For psychological stress detection, the proposed feature selection method can achieve better performance compared to conventional methods with fewer ECG features or BVP features. The feature selection method proposed in this paper has great application prospects in the field of psychological stress detection because this method can allow the model to achieve good performance with fewer features.

## 5. Conclusion

In this paper, a novel feature selection method for stress detection was proposed. An emotion-evoking experiment was conducted to induce emotions with three valence levels from participants. In the emotion-evoking experiment, participants were presented with three groups of images with different valence levels. ECG, BVP, and GSR signals were collected to train the four classifiers (KNN, DT, RF, and SVM) in different classification tasks. For feature selection, 13 features were selected by the proposed method, 17 features were selected by the PCA (M2), and 22 features were selected by M1. The results in Table 2 showed that the proposed method can achieve the highest accuracy when three physiological signals are used for classification, the proposed method can achieve 0.988 accuracy in three emotions recognition and can achieve 0.991 accuracy in stress detection. The results of this study confirmed the effectiveness of the proposed method. Our study also indicated that the analysis of physiological signals during emotional change is helpful to find sensitive features. In general, the novel feature selection method proposed by this paper will reduce the obstacles for the construction of an efficient healthcare emotion recognition model. In the future research work, we will deploy the proposed emotion recognition model to the hardware platform, and further improve our model according to the actual needs of healthcare.

**Table 2:** Classification performance of different physiological signal combinations.

**Different combinations**	**Emotion recognition**	**Stress detection**
ECG	0.887	0.913
BVP	0.926	0.951
GSR	0.960	0.970
ECG&BVP	0.962	0.984
ECG&GSR	0.974	0.989
BVP&GSR	0.977	0.990
ECG&BVP&GSR	0.988	0.991

## Data availability statement

The raw data supporting the conclusions of this article will be made available by the authors, without undue reservation.

## Ethics statement

The studies involving human participants were reviewed and approved by Scientific Research Ethics Committee of the General Hospital of the Southern Theater Command of the Chinese People's Liberation Army. The patients/participants provided their written informed consent to participate in this study.

## Author contributions

YX proposed the idea and conducted the experiments. ZL wrote the manuscript. ZL and LZ provided important advice on the research approaches, signal processing, and checked and revised the manuscript. YP and MJ offered important help that guided the experiments and analysis methods. All authors contributed to the article and approved the submitted version.
